# The Association of −330 Interleukin-2 Gene Polymorphism with Its Plasma Concentration in Iranian Multiple Sclerosis Patients

**DOI:** 10.1155/2014/724653

**Published:** 2014-05-19

**Authors:** Arezou Sayad, Abolfazl Movafagh

**Affiliations:** Department of Medical Genetics, Shahid Beheshti University of Medical Sciences, Tehran 1985717443, Iran

## Abstract

Multiple sclerosis (MS) is a chronic neuroinflammatory demyelinating disease of the central nervous system. The cytokine genes are involved in autoimmune diseases such as MS. In this study, we report the influence of −330 interleukin-2 (IL2) gene polymorphism on its plasma levels in a group of Iranian MS patients. In this study 100 MS patients and 100 ethnically, age, and sex matched healthy controls were selected from Medical Genetics Department of Sarem Women Hospital. Blood samples of all individuals were collected in EDTA tubes. The restriction fragment length polymorphism PCR (RFLP) method was applied to determine various alleles and genotypes in these individuals. Plasma concentration of IL2 was measured in all the samples using human IL2 kit. The frequency of −330 T/T IL2 genotype was higher in MS patients compared to normal individuals. Accordingly, the plasma levels of IL2 were significantly higher (*P* < 0.0001) in patients when compared to the control group. In conclusion, in case of MS patients the −330 T/T IL2 genotype is associated with higher plasma levels of IL2.

## 1. Introduction

Multiple sclerosis (MS) is defined as a sort of chronic neuroinflammatory and autoimmune disorder according to demyelization of the central nervous system. It is supposed to be initiated and regulated by autoreactive T cells directed against myelin antigens. Both genetic and environmental elements contributed to disorder risk [1].

Interleukin-2 (IL2) is defined as a cytokine involved in the operation and regulation of immune system. IL2 is regarded as pro- and anti-inflammatory element. IL2 was recognized as an autocrine secretary product from activated T cells with growth factor characteristics. It has been reported that IL2 elicits T-cell proliferation, survival, and differentiation of effectors. The IL2 gene is a significant functional factor which is involved in immune regulation and function. The main function of IL2 is the maintenance of peripheral T-cell tolerance; it has a vital role in regulatory T-cell (T-reg) homeostasis. The impairment of T-reg cells is probably responsible for the autoimmunity in the absence of IL2 [2, 3].

Several lines of studies suggest that IL2 is implicated in the pathogenesis of MS [4–10]. It has been demonstrated that the concentration of IL2 increases in cerebrospinal fluid (CSF) as well as in sera of MS patients [11]. John and colleagues (1998) detected the −330 IL2 promoter single nucleotide polymorphism [12]. Matesanz et al. showed that both of −330 G/T and T/T genotypes of IL2 gene have an association with susceptibility to secondary progressive (SP) course of MS [9]. They indicated that IL2 promoter luciferase constructs transected in Jurkat cell line showed higher significant levels of gene expression in −330 G allele. In contrast, they found IL2 expression in lymphocyte increased in carriers of G/T and T/T genotype. Previously, we reported various polymorphisms of IL2 gene [13, 14] and other genes [15–17] which are involved in immune system in autoimmune diseases. However, to our knowledge impact of −330 IL2 polymorphism on the plasma concentration or expression of IL2 in MS patients among ethnic groups has not been reported.

In the present study, we report the impact of the −330 IL2 gene polymorphism (rs 2069762) on the IL2 concentration in plasma samples of MS patients.

## 2. Materials and Methods

### 2.1. Subject and Control Groups

One hundred distinct patients with relapsing remitting MS were selected in this study. The patients had mean standard deviation age of 27 ± 5.9 years and age range of 20–42 years. These patients were selected from Medical Genetics Department of Sarem Women Hospital and diagnosed by neurologist based on the McDonald criteria [18]. Besides, 100 ethnically, age, and sex matched healthy individuals with no personal or family history of autoimmune diseases were selected. Control group had mean age of 29.8 ± 7.8 years and age range of 20–52 years. All controls were informed of the research and gave their full written consent.

### 2.2. Data Extraction and Genotyping

Five mL of blood sample was collected from each individual in EDTA tube and plasma isolated. DNA was extracted from peripheral blood samples by salting out method [19]. Then DNA sample was subjected to restriction fragment length polymorphism PCR (RFLP). 100 nanograms of extracted DNA were amplified using specific primers: forward 5′- ATTCACATGTTCAGTGTAGTTCT-3′ and reverse 5′-GTGATAGCTCTAATTCATGC-3′. The PCR conditions were 94°C for 4 min followed by 35 cycles of 20 s at 94°C, 40 s at 52°C, 20 s at 72°C, and a final prolongation step of 10 min at 72°C. The PCR amplification yielded a band of 131 bp. After digestion by Bfa-1 restriction enzyme (New England Biolabs), the PCR products were digested to 110 and 21 bp fragments. 12% polyacrylamide gel electrophoresis was used.

Plasma concentration of IL2 was detected using a Human IL2 kit purchased from eBioscience company (http://www.eBioscience.com). The kit was used according to its manufacturer's instruction. Finally, the comparison between −330 IL2 genotype and IL2 plasma level was done.

### 2.3. Statistical Analysis

To examine the effect of −330 IL2 polymorphism and plasma concentration of IL2, independent Student's *t*-test was performed. Differences between the parameters measured in patients and control group were compared, and *P* value <0.05 was considered significant. SPSS 18v for windows software was utilized.

## 3. Result

**Figure 1:**
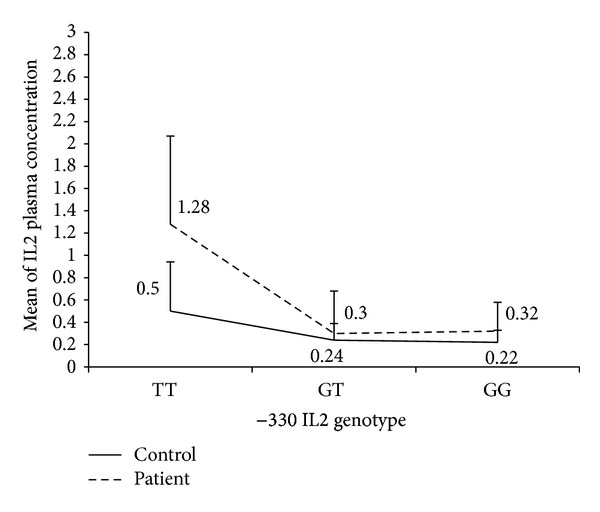
Plasma concentration of IL2 in MS patients with −330 T/T IL2 genotype compared to controls.

The frequency of the T allele at the −330 IL2 polymorphism was significantly higher in patients than controls (OR: 1.88, 95% CI: 1.26–2.79, and *P* = 0.002). Moreover, the T/T genotype was more frequent in patients than in control (42% versus 28%, OR: 1.86, 95% CI: 1.03–3.36, and *P* = 0.03) (Table 1). The influence of −330 IL2 gene polymorphism on plasma concentration of IL2 was shown in Table 2. The comparisons between genotypes in each MS and control group were shown in Table 3. The patients who carried T/T genotype had higher plasma concentration of IL2 compared to that measured in controls (*P* < 0.0001). The plasma concentration of IL2 was significantly higher (2.5-fold) in carriers of homozygote T genotype.  Figure 1 showed that IL2 concentration was higher in patients than in controls, but only patients who carried T/T genotype had increased amount of IL2 significantly.

## 4. Discussion

The role of IL2 in maintenance of the self-tolerance and its activity in the main nerve system may have an effect on autoimmune diseases such as MS. Therefore different −330 IL2 genotypes may influence plasma concentration of IL2 in MS patients.

In this work, the impact of the −330 IL2 polymorphism on the concentration of IL2 between MS patients and healthy controls was investigated. This study demonstrated that the −330 T/T genotype which was significantly more frequent in MS patients was related to a higher level of IL2 plasma concentration in comparison to controls. Matesanz and colleagues evaluated the expression of the −330 IL2 and T alleles in vivo and in vitro [10]. Their work, which was performed on Jurkat cell line, demonstrated an unlikely promoter relation between the G and T alleles. The promoter with the G allele was twice more effective than the one with the T allele. On the contrary, quantification of allelic expression in lymphocytes showed that the −330 T allele was associated to a higher level of transcription than the −330 G allele. Moreover, they showed higher level of IL2 mRNA expression in cases of individuals with −330 T/T and G/T genotypes than controls with the −330 G/G genotype. These results were not in accordance with those of Hoffman and colleagues who described an increase in IL2 production in controls genotyped as −330 G/G [20]. Matesanz et al. proposed that the distinction between the in vitro and in vivo effect of the −330 IL2 promoter polymorphic locations indicated the existence of additional unknown polymorphisms that affected gene adjustment [10].

Moreover, the comparison of IL2 concentration between carriers of T/T versus G/G genotypes and also T/T versus G/T in the control group demonstrated that there are significant differences between them (*P* = 0.0004 and *P* = 0.0013, resp.) and in the MS patients group too (*P* = 0.0001 and *P* = 0.0001, resp.). Indeed, −330 T/T genotype of IL2 gene may have an effect on increasing IL2 plasma level in general population (e.g., control group). Since carriers of T/T genotype in the MS patients group have significantly higher IL2 concentration in comparison to healthy controls, it seems that maybe there are additional mechanisms that affect or control the relation of T/T genotypes and IL2 concentration in MS patients.

In this study, we investigated the association of the T/T genotype at the −330 position of the IL2 gene with the plasma levels of IL2 in Iranian MS patients. We observed that this effect is significantly higher in MS patients than in healthy controls. It shows there would be relation between this SNP and concentration of IL2 in MS patients. It seems that studies with large sample size are required to bring about more authentic results.

## 5. Conclusion

In conclusion, the patients who carried T/T genotype exhibited higher plasma levels of IL2.

## Figures and Tables

**Table 1:** Frequencies of −330 IL2 alleles and genotypes in patients and controls.

	Patients (%)	Control (%)	OR	CI (95%)	*P*
Allele	*n* = 200	*n* = 200	1.88	(1.26–2.79)	0.002
G	78 (39%)	109 (54.5%)			
T	122 (61%)	91 (45.5%)			
Genotype	*N* = 100	*N* = 100			
G/G	20 (20%)	37 (37%)	0.426	(0.225–0.804)	0.008
G/T	38 (38%)	35 (35%)	1.138	(0.640–2.025)	0.659
T/T	42 (42%)	28 (28%)	1.862	(1.032–3.360)	0.038

**Table 2:** The influence of −330 IL2 gene polymorphism on plasma concentration of IL2.

Genotype	Patients (*N* = 100)	Controls (*N* = 100)	*P*
	Mean concentration of IL-2 ± SD (*N*)	Mean concentration of IL-2 ± SD (*N*)	
G/G	0.32 ± 0.26 (20)	0.221 ± 0.108 (37)	0.051
G/T	0.3 ± 0.382 (38)	0.24 ± 0.149 (35)	0.329
T/T	1.288 ± 0.795 (42)	0.5 ± 0.44 (28)	<0.0001

**Table 3:** The comparisons between genotypes in each MS and control group.

Genotypes	Patients (*N* = 100)		Controls (*N* = 100)	
	Mean concentration of IL-2 ± SD (*N*)	*P* value	Mean concentration of IL-2 ± SD (*N*)	*P* value
G/G versus T/T	0.32 ± 0.26 (20) versus 1.288 ± 0.795 (42)	0.0001	0.221 ± 0.108 (37) versus 0.5 ± 0.44 (28)	0.0004
G/G versus G/T	0.32 ± 0.26 (20) versus 0.3 ± 0.382 (38)	0.83	0.221 ± 0.108 (37) versus 0.24 ± 0.149 (35)	0.53
T/T versus G/T	1.288 ± 0.795 (42) versus 0.3 ± 0.382 (38)	0.0001	0.5 ± 0.44 (28) versus 0.24 ± 0.149 (35)	0.0013
